# Health of Large Cities

**Published:** 1858-04

**Authors:** 


### HEALTH OF LARGE CITIES.

TWENTY-FIVE persons out of every thousand die in London every year. In Philadelphia, twenty; in New York, a fair estimate is, about thirty-four in a thousand. This comparison seems to place New York in an unfavorable light, and does not do her justice; for it is well known that thousands die here who have contracted their diseases in European jails, poor-houses, and hospitals; or by penury, drunkenness, hard-living, and idleness, and reach our shores but to die. In less than four months, ending May 14, 1857, one Episcopal clergyman of our city buried six persons, whose united ages were *four hundred and eighty years*, averaging full four-score—all born in the city but one, and that one in the northern suburbs; all of them attended public worship, and two-thirds were regular communicants: showing, certainly, that "*length of days is in their right hand*" who habitually worship the God of their fathers, and whose declining years are passed under the soothing influences of religious contemplations. Let all, then, who wish to live long, begin early to arrange their affairs, so that they may have quietude of mind, and that rest for the body which will afford leisure for the cultivation of religious feelings, and the contemplation of Divine truths.

